# Impact of herbicide-resistant genetically modified rapeseed on gut bacterial diversity of *Eisenia fetida*

**DOI:** 10.1080/21645698.2026.2617700

**Published:** 2026-01-20

**Authors:** Ye-Jin Jang, Sung-Dug Oh, Joon Ki Hong, Na-Yeon Kim, Gyeong-Min Lee, Soo-Yun Park, Jong-Chan Park, Ancheol Chang

**Affiliations:** a Department of Agricultural Biotechnology, National Institute of Agricultural Sciences, Rural Development Administration, Jeonju, Korea; b Technology Cooperation Bureau, Rural Development Administration, Jeonju, Korea

**Keywords:** *Eisenia fetida*, environmental risks, GM rapeseed, gut bacterial diversity, horizontal gene transfer

## Abstract

The systematic evaluation of the safety and environmental impact associated with genetically modified (GM) crops is currently underway within the scientific community, with a particular focus on their effects on the gut microbiota, which plays a vital role in host health. In this study, we compared the effects of a non-GM rapeseed cultivar with those of an herbicide-resistant GM rapeseed cultivar containing the phosphinothricin acetyltransferase gene on the gut bacterial community of *Eisenia fetida*. The 16S rRNA amplicon sequencing and data analysis showed no significant differences in gut bacterial community composition or diversity between *E. fetida* fed GM rapeseed and those fed non-GM rapeseed. Principal component analysis indicated that, rather than plant type, external factors influenced the community structure. Polymerase chain reaction analysis revealed no evidence of horizontal gene transfer from GM rapeseed to microbes or earthworms. Overall, GM rapeseed had a negligible effect on gut microorganisms and did not significantly alter the gut bacterial community of *E. fetida*.

## Introduction

Globally, the commercial cultivation of genetically modified (GM) crops has spread rapidly since 1996, with more than 209.8 million hectares of GM crops planted in 27 countries in 2024.^1^ Many of these GM crops possess insect resistance and herbicide tolerance, and have provided a range of economic benefits, including increased agricultural productivity and reduced farming costs.^2^ Among them, rapeseed (*Brassica napus* L.) has attracted attention as a source of high-quality edible oil, and efforts have been made to address weed control through the development of herbicide-tolerant varieties.^3^

Traditional safety assessment of GM crops has been centered on short-term toxicology experiments, including analyses of body weight changes as well as blood and organ markers; however, the impact of GM crops on the gut microbiome has recently gained attention as a new endpoint.^4^ The gut microbiota plays a key role in host immunity, metabolism, and nutrient absorption, and the gut is considered one of the largest immune organs *in vivo*.^5,6^ Various studies have raised the possibility that consumption of GM crops may affect gut microbial diversity, and some have evaluated the effects of GM maize on the gut microbiota composition of mammals.^7,8^ However, most studies have focused on farmed animals, and there is a relative lack of research on native decomposers, which are key organisms in soil ecosystems.

Earthworms (*Eisenia fetida*) are representative decomposers of animal and plant residues in soil ecosystems. They contribute to organic matter decomposition and nutrient cycling and exhibit sensitive responses to environmental toxicants through changes in their gut microbiota.^9,10^ As representative soil macro-organisms that maintain essential ecosystem functions – including soil aggregation, nutrient cycling, and the promotion of plant growth – earthworms are frequently utilized as key indicators to evaluate soil health and environmental risks.^10^ Therefore, *E. fetida* is considered a useful bioindicator species in the ecotoxicological assessment of GM crops. Many ecological functions of earthworms are related to their gut microbiota,^10^ however, to date, few studies have analyzed the effects of herbicide-tolerant GM crops on the gut bacterial diversity of *E. fetida*.

The persistence of foreign genes and proteins in the soil, originating from root secretions or residues of GM crops, can induce structural changes in the soil microbial community. Such alterations can exert broad ecological impacts on various soil-dwelling organisms, with particularly significant indirect effects on the gut microbiota of soil macro-invertebrates such as earthworms, which play a key role in processing soil organic matter.^11,12^ Therefore, determining the effects of GM crops on soil organisms, either directly or indirectly, is essential for the ecological safety assessment of GM crops.

The GM rapeseed used in this study is *Brassica napus* cv. “Yongsan,” which possesses the *BrAGL20* gene, a major integration gene that regulates flower development. The gene was introduced through an *Agrobacterium*-mediated transformation method.^13^ This GM rapeseed contains the *bar* gene, which confers herbicide tolerance by expressing the phosphinothricin acetyltransferase (*pat*) gene, an enzyme that detoxifies glufosinate herbicides. GM rapeseed is widely used by major producers, such as Canada, the USA, and Australia; however, some countries require the safety evaluation of individual GM events. This necessitates an assessment of the toxicological and gut microbiological safety of individual GM varieties.

In this study, herbicide-tolerant transgenic rapeseed was fed to *E. fetida*, and its effects on the gut bacterial diversity and community structure of *E. fetida* were assessed using 16S rRNA-based metagenomic analysis. This study aimed to characterize the long-term and detailed effects of GM crops on non-target organisms and to provide a basis for future environmental risk assessments and safety standards.

## Materials and Methods

### Plant Material

**Figure 1.**
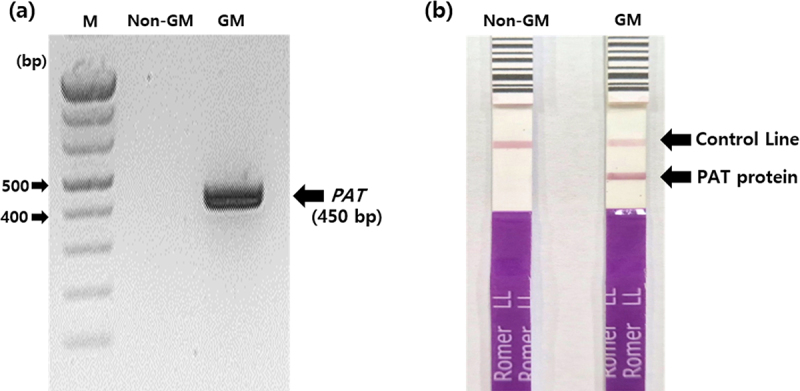
Genetic characterization of herbicide-resistant transgenic rapeseed (GM). (a) Confirmation of the presence of the *pat* gene by polymerase chain reaction; (b) detection of PAT protein expression using the immunostrip assay. M, DNA ladder (D1037, Bioneer, Korea); non-GM, non-GM rapeseed cultivar “Yongsan”; GM, herbicide-resistant transgenic rapeseed. PAT, phosphinothricin acetyltransferase.

In a previous study, herbicide-resistant transgenic rapeseed (GM rapeseed) was developed through *Agrobacterium*-mediated transformation of a non-GM rapeseed variety (*Brassica napus* cv. “Youngsan”).^13^ The GM rapeseed used in this study was provided by Dr. Hong at the National Institute of Agricultural Sciences, Rural Development Administration. The presence of the transgene was confirmed using polymerase chain reaction (PCR) and immunostrip analysis (Figure 1). Rapeseed leaves were frozen in liquid nitrogen, and genomic DNA was extracted using a DNeasy Plant Kit (Qiagen, Hilden, Germany). PCR was performed using the StepOnePlus™ Real-Time PCR system (Fisher Scientific, New Hampshire, USA) under the following conditions: initial denaturation at 95°C for 3 min, followed by 35 cycles of denaturation at 95°C for 30 s, annealing at 58°C for 30 s, extension at 72°C for 30 s, with a final extension at 72°C for 5 min. A set of primers (F: 5”-TCTGCACCATCGTCAACCACTACAT-3‘; R: 5’-CTGAAGTCCAGCTGCCAGAAA CCC A-3”) that specifically amplify the *pat* gene was used for PCR. For the immunostrip test, samples were treated with an extraction solution to isolate proteins, and PAT protein expression was analyzed using the SeedChek® LL Test Strip (Romerlabs, Getzersdorf, Austria). The cultivation of non-GM and GM rapeseed was carried out in Rural Development Administration GMO-isolated fields (RDA-AB-2013–041) located in Jeonju, Jeollabuk-do, Korea. Whole GM and non-GM rapeseed plants were sampled after flowering and used as food for earthworms. The collected samples were thoroughly dried using a freeze-dryer (HyperCool-HC8080; LABOGENE, Gimpo, Korea), and the dried samples were powdered using a Planetary Ball Mill (PM100; Fritsch, Baden-Württemberg, Germany) at 350 rpm for 5 min.

### Earthworms (E. fetida) Rearing Conditions

The artificial soil for earthworm cultivation was prepared by mixing sand (Biotope Rio Tefe 0.1–0.2 mm, Nature Farm, Seoul, Korea), kaolin (Kaolin JP-100, BuyChemJapan, Osaka, Japan), and peat moss (Sphagnum peat moss, Berger, Quebec, Canada) in a 7:2:1 ratio, with a total of 555 g used per beaker. The prepared soil mixture was stabilized at room temperature for 24 h before use to ensure uniform consistency. Earthworms (*Eisenia fetida*) were maintained at 20°C under continuous dark conditions (24 h) throughout the experiment. Each earthworm was fed 3 g of Magic Worm Food (Magic Products Inc., Wisconsin, USA) once a week. The experimental design consisted of three distinct groups: a control group receiving only the standardized basal diet (Magic Worm Food), and two treatment groups supplemented with either non-GM rapeseed powder or 0.1% (w/w) herbicide-tolerant GM rapeseed powder. The concentration of 0.1% (w/w) was determined through preliminary trials to prevent alterations in soil texture and physical structure that could induce nonspecific stress in *E. fetida*. For experimental use, only adult earthworms weighing > 300 mg were selected, while those weighing > 600 mg were excluded to maintain a homogeneous population. Earthworms were sorted by body weight and placed in separate beakers, with ten earthworms per 2 L beaker (Ø135 mm × 200 mm, Dongsung Science, Seoul, Korea), and each treatment was replicated three times. From each replicate, three earthworms were randomly selected for gut microbiota analysis. The experiment was conducted over 112 weeks, which was confirmed as the average lifespan of *E. fetida* in a preliminary trial, to evaluate the effects of the sample treatments on earthworms and soil properties. Treatment samples were applied at a concentration of 0.1% (w/w) based on the total soil weight. Feed and treatments were administered at 4-week intervals. Earthworm weight, soil pH, and soil moisture content were measured at 4-week intervals to monitor biological and environmental changes throughout the study. Soil moisture content was determined using a high-precision moisture analyzer (XC63, CAS, Yangju, Korea), and distilled water was added when soil moisture fell below 40% to maintain optimal humidity.

### DNA Extraction, PCR Amplification, and 16S rRNA Gene Sequencing

The surface of each earthworm was thoroughly sterilized with 70% ethanol to remove adhering soil particles and transient microorganisms. All surgical instruments used for dissection were also disinfected with 70% ethanol before each use to prevent cross-contamination. Following surface sterilization, the gut was carefully dissected and separated from the surrounding body wall tissue under sterile conditions to ensure the integrity of the internal microbial samples. Total genomic DNA was extracted from the samples using the DNeasy Blood & Tissue Kit (Qiagen, Hilden, USA), following the manufacturer’s protocol. The V3–V4 hypervariable regions of the bacterial 16S rRNA gene were amplified using fusion primers 341F (5′-AATGATACGGCGACCACCGAGATCTACACXXXXXXXXTCGTCGGCAGC-GTCAGATGTGTATAAGAGACAGCCTACGGGNGGCWGCAG-3’) and 805 R (5′-CAAG-CAGAAGACGGCATACGAGATXXXXXXXXGTCTCGTGGGCTCGGAGATGTGTATA-AGAGACAGGACTACHVGGGTATCTAATCC-3′), incorporating Illumina adapter sequences, dual indices, and Nextera transposase sequences. PCR was performed under the following cycling conditions: initial denaturation at 95°C for 3 min; 25 cycles of denaturation at 95°C for 30 s, annealing 55°C for 30 s, and extension at 72°C for 30 s; followed by a final extension at 72°C for 5 min. The amplified products were confirmed by 1% agarose gel electrophoresis and purified using a magnetic bead – based cleanup. Short nonspecific fragments were removed using the ProNex® Size-Selective Purification System (Promega, Madison, WI, USA). DNA concentration and quality were assessed using the QuantiFluor^TM^ dsDNA System (Promega, Madison, WI, USA). Equimolar concentrations of the purified amplicons were pooled and subjected to paired-end sequencing (2 × 300 bp) using the Illumina MiSeq platform at CJ Bioscience Inc. (Seoul, Korea).^12^

### Bioinformatic Processing and Microbial Community Analysis

Raw sequencing reads were processed using Trimmomatic v0.32 to remove low-quality bases (Q < 25). Quality-filtered paired-end reads were merged using the fastq_mergepairs function in VSEARCH v2.13.4 under default settings. Primer sequences were trimmed using the Myers – Miller alignment algorithm with a similarity threshold of 0.8. Nonspecific amplicons lacking 16S rRNA gene signatures were identified and excluded using nhmmer from the HMMER v3.2.1 package with 16S-specific hidden Markov model (HMM) profiles. Dereplication was performed using the derep_full-length command in VSEARCH to identify unique sequences and group redundant reads. Taxonomic assignments were conducted using the EzBioCloud 16S rRNA reference database with the usearch_global command, followed by refined pairwise alignment. Chimeric sequences were removed using the UCHIME algorithm with a reference-based approach against the EzBioCloud non-chimeric 16S database. Sequences that were not assigned at the species level ( < 97% similarity) were further clustered *de novo* using the cluster_fast command to generate additional operational taxonomic units (OTUs). OTUs represented by singleton reads were excluded from downstream analyses. Secondary analyses were performed using the EzBioCloud 16S-based MTP platform (CJ Bioscience, Seoul, Korea). Alpha diversity metrics, including ACE, Chao1, Jackknife, Shannon, NPShannon, Simpson, and Phylogenetic Diversity, were calculated, and rarefaction and rank abundance curves were generated. Beta diversity was assessed using multiple distance algorithms, including Jensen – Shannon, Bray – Curtis, Generalized UniFrac, and Fast UniFrac. Functional profiles were predicted using PICRUSt and MinPath, and both taxonomic and functional biomarkers were identified using Linear Discriminant Analysis Effect Size and the Kruskal – Wallis H test.^12^

### Analysis of Horizontal Gene Transfer (HGT)

To assess the potential for horizontal gene transfer from transgenic rapeseed, the *bar* gene was analyzed in earthworm-treated soil and earthworm body samples. Genomic DNA was extracted from soil and earthworm body samples using a DNeasy Blood & Tissue Kit (Qiagen, Hilden, USA) according to the manufacturer’s instructions. PCR amplification was performed using primer pairs that specifically amplify the *bar* gene introduced into GM rapeseed. Initial denaturation was performed at 95°C for 5 min, followed by 30 cycles of denaturation (95°C, 30 s), annealing (58°C, 30 s), and elongation (72°C, 30 s), with a final elongation at 72°C for 5 min. To ensure the reliability of the HGT analysis and to exclude the possibility of false-negative results, the absence of the transgene was cross-verified using multiple primers specific to the promoter and terminator regions, with positive controls included in all PCR assays. The PCR products were analyzed using 1% agarose gel electrophoresis. To confirm the quality of soil microbial DNA, PCR was performed under the same conditions using universal primers (27F/1492 R) targeting the 16S rRNA gene. Earthworm body DNA quality was verified by targeting the *β-actin* gene.

### Statistical Analysis

All statistical analyses were conducted using SPSS Statistics (23.0.0 for Windows, Rel.23.0, 2015.2 SPSS Inc., Chicago, USA). Differences between means were tested using Duncan’s test and analysis of variance with a significance threshold of *p* < .05. Heatmap visualization of microbial abundance was performed using Python (v3.10, Python Software Foundation, Beaverton, OR, USA) with the seaborn (v0.12) and matplotlib (v3.7) libraries.

## Results

### Effect of GM Rapeseed on the Growth of Earthworms (E. fetida)

**Figure 2.**
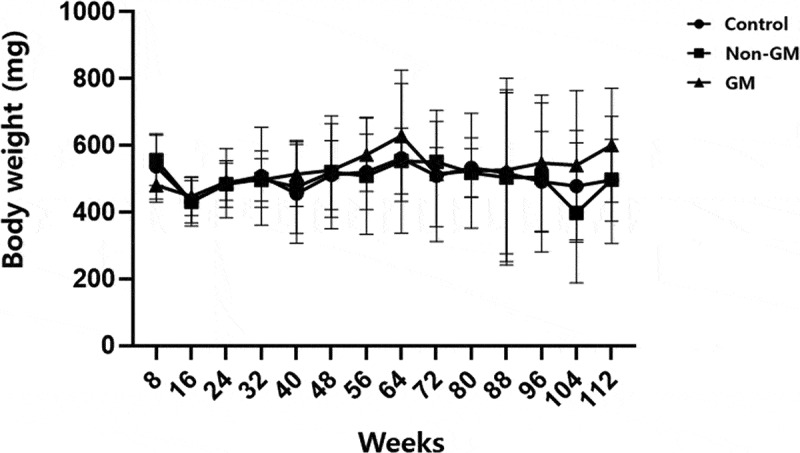
Body weight changes of *E. fetida* fed different rapeseed varieties. Control, the group fed with standard feed only; non-GM, the group fed with the non-GM rapeseed cultivar “Yongsan”; GM, the group fed with herbicide-resistant transgenic rapeseed. Error bars represent standard errors. GM, genetically modified.

When comparing the weight changes among earthworms fed GM rapeseed, controls, and earthworms fed non-GM rapeseed groups at 4-week intervals, no significant differences in growth were observed among the three groups. Throughout the experimental period, all groups exhibited similar weight gain, indicating that feeding with GM rapeseed did not adversely affect earthworm growth (Figure 2). Furthermore, long-term monitoring revealed that the first mortality in all groups occurred at 92 weeks, likely due to natural aging, as the earthworms had reached their average lifespan. These findings suggest that feeding on GM rapeseed does not negatively affect earthworm survival.

### Microbial Community Analysis in the Rhizosphere

**Figure 3.**
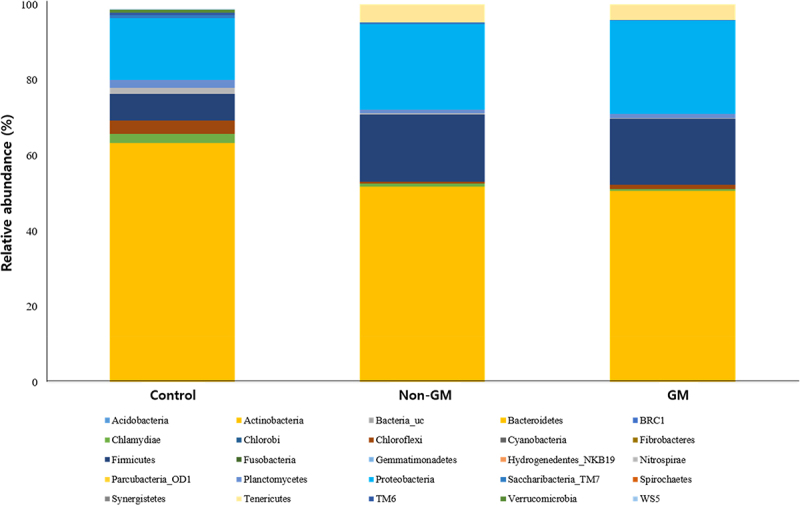
Comparison of gut bacterial composition at the phylum level of *E. fetida*. Control, the group fed with standard feed only; non-GM, the group fed with the non-GM rapeseed cultivar “Yongsan”; GM, the group fed with herbicide-resistant transgenic rapeseed. GM, genetically modified; *E. fetida, Eisenia fetida.*

A total of 29 bacterial phyla were identified through gut microbiota analysis of earthworms fed with GM and non-GM rapeseed (Figure 3). To evaluate the impact of GM rapeseed on gut microbial community composition, the relative abundance of each phylum was compared. Actinobacteria were the most dominant phylum across all groups, accounting for 62.7%, 50.0%, and 50.1% of phyla identified in the control, non-GM, and GM groups, respectively. Gemmatimonadetes and Chloroflexi were present at low levels, ranging from 0.01–0.03% and 0.5–3.4%, respectively. Firmicutes showed increased abundance in both the non-GM (17.9%) and GM (17.6%) groups relative to the control (7.1%). Acidobacteria exhibited a relatively high abundance in the GM group (1.2%), although it remained low overall. These results suggest that feeding earthworms with GM rapeseed does not significantly alter the overall gut microbial community structure.

**Figure 4.**
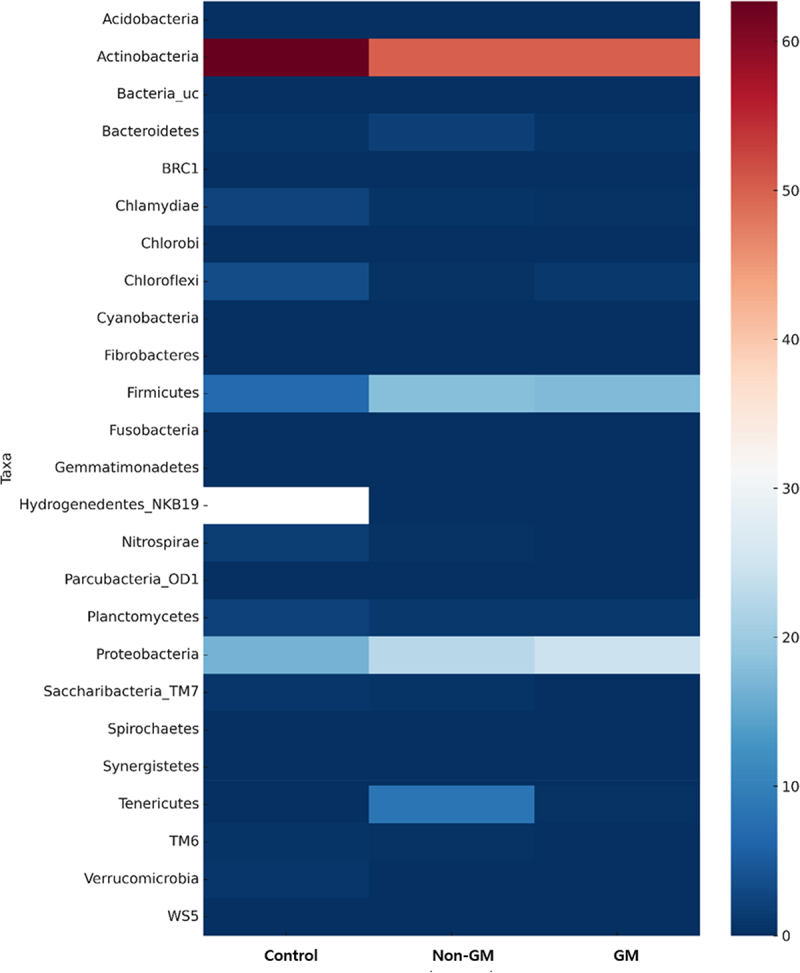
Heatmap visualizing the relative abundance of OTU levels in each experimental group. Color indicates the magnitude of abundance, with changes from blue to red indicating higher relative abundance. Control, the group fed with standard feed only; non-GM, the group fed with the non-GM rapeseed cultivar “Yongsan”; GM, the group fed with herbicide-resistant transgenic rapeseed. GM, genetically modified; OTU, operational taxonomic unit.

To assess the differences in microbial community composition among the three groups, a heatmap was generated based on the relative abundance of OTUs (Figure 4). The analysis revealed that both the GM and non-GM groups exhibited similar overall microbial community patterns compared to the control group, with only minor variations observed in specific OTUs. The major taxonomic groups – Actinobacteria, Firmicutes, and Bacteroidetes – were relatively abundant across all conditions. Notably, Actinobacteria maintained nearly identical abundance levels in both the control and treatment groups. In contrast, Bacteroidetes showed a trend toward increased abundance in both the GM and non-GM groups relative to the control; however, no significant differences were detected between the GM and non-GM groups, suggesting that the effect of GM treatment alone was limited. Some OTUs (e.g., Acidobacteria, *Bacteria_uc*) exhibited a slight increase in abundance under both GM and non-GM conditions; however, no significant differences were observed between the two treatment groups. Overall, meaningful shifts in the OTU distribution were evident only when comparing the GM and non-GM groups with the control. Low-abundance OTUs such as Armatimonadetes and Chloroflexi remained consistently scarce across all groups, indicating that they were relatively inactive under the experimental conditions. These findings suggest that microbial community shifts associated with GM treatment are essentially similar to those observed under non-GM conditions. The changes in community composition observed in both treatment groups appeared to stem primarily from differences relative to the control group. This implies that external environmental factors may have had a greater influence on microbiome structure than the GM status of the crop itself.

**Figure 5.**
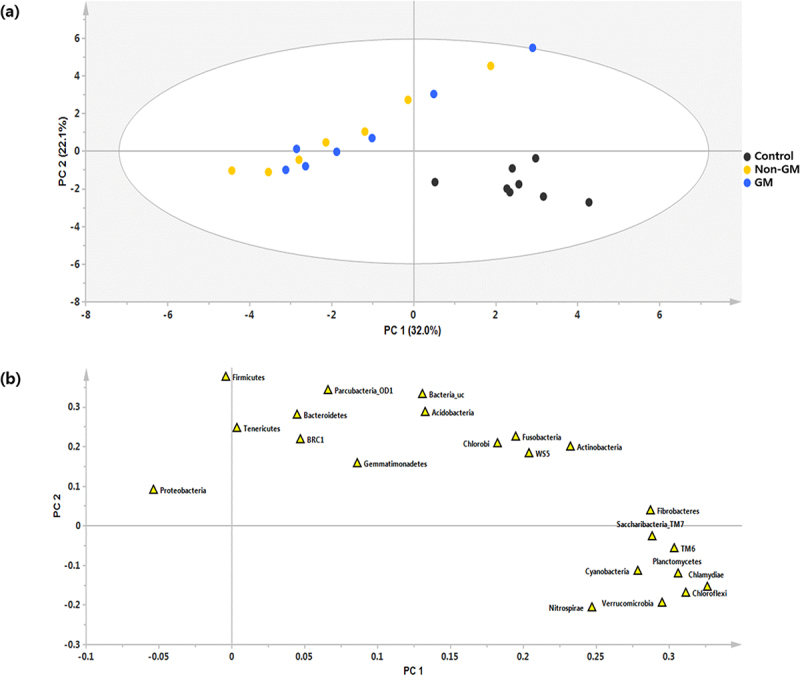
PLS-DA score and loading plots showing gut microbial community structure and OTU contributions among experimental groups. Control, the group fed with standard feed only; non-GM, the group fed with the non-GM rapeseed cultivar “Yongsan”; GM, the group fed with herbicide-resistant transgenic rapeseed. GM, genetically modified; OTU, operational taxonomic unit; PLS-DA, partial least squares discriminant analysis.

Partial least squares discriminant analysis revealed distinct microbial community structures among the three groups, as shown in the score plot (Figure 5a), and identified the major contributing OTUs in the loading plot (Figure 5b). The control group was clearly separated from the GM and non-GM groups along PC1, indicating significant differences in community composition. However, GM and non-GM groups clustered closely, suggesting that external factors or treatment conditions, rather than GM treatment alone, primarily drove the observed changes.

### Alpha Diversity of Bacterial Communities

**Figure 6.**
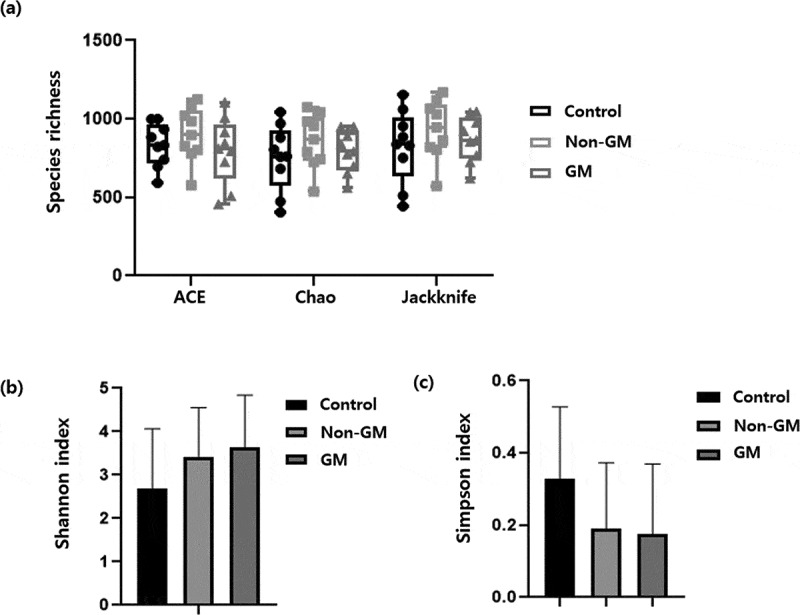
Comparison of alpha diversity indices of gut microbial communities in *E. fetida* among control, non-GM, and GM rapeseed groups. Control, the group fed with standard feed only; non-GM, the group fed with the non-GM rapeseed cultivar “Yongsan”; GM, the group fed with herbicide-resistant transgenic rapeseed. GM rapeseed. GM, genetically modified; *E. fetida, Eisenia fetida.*

Alpha diversity indices indicated no significant differences in microbial richness or diversity among the three groups (Figure 6). The ACE index, which estimates species richness, showed comparable values across the control, non-GM, and GM rapeseed groups, with no significant differences (Figure 6a). Similarly, the Shannon diversity index, which reflects both richness and evenness, revealed slightly higher mean values in the non-GM and GM groups than in the control group; however, these differences were not significant due to large variation among replicates (Figure 6b). The Simpson index, which emphasizes the dominance of certain taxa, also showed no significant differences among the groups, although the control group exhibited a slightly higher average value (Figure 6c). These results suggest that the introduction of GM rapeseed did not significantly affect the alpha diversity of microbial communities relative to the non-GM and control conditions. This implies a stable community structure under the tested environmental conditions.

### Horizontal Gene Transfer

**Figure 7.**
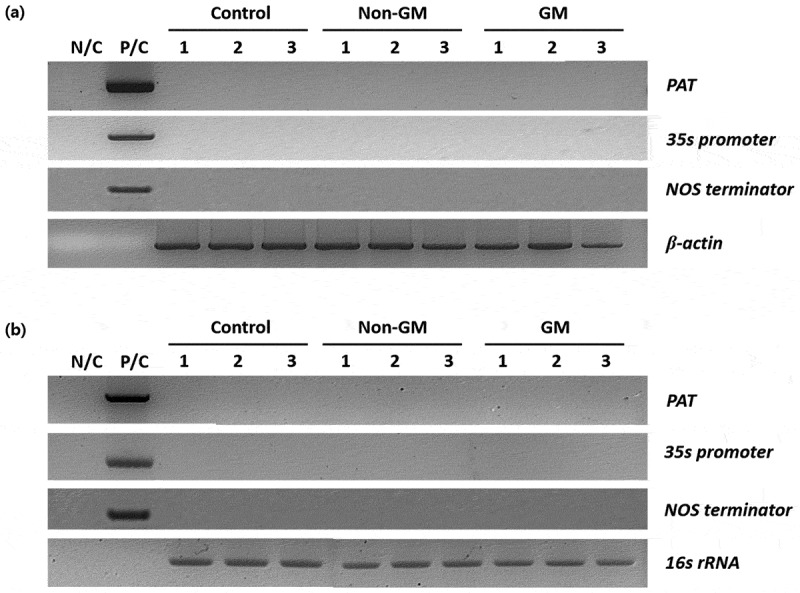
Detection of the *pat* gene in earthworms and rearing soil after feeding with GM rapeseed. (a) PCR analysis of DNA extracted from the bodies of earthworms fed with GM rapeseed; (b) PCR analysis of DNA extracted from the rhizosphere soil in which the earthworms were reared. N/C, negative control (no DNA); P/C, positive control (genomic DNA of GM rapeseed); control, the group fed with standard feed only; non-GM, the group fed with the non-GM rapeseed cultivar “Yongsan”; GM, the group fed with herbicide-resistant transgenic rapeseed. GM, genetically modified; *pat*, phosphinothricin acetyltransferase gene; PCR, polymerase chain reaction.

To assess the environmental risk of GM rapeseed, PCR analysis was performed to examine whether the inserted gene was horizontally transferred to soil microorganisms or to the test organism. In this study, we investigated the presence of the *pat* gene – inserted into the GM rapeseed genome – in the bodies of earthworms and in the soil microbial community where the earthworms were reared after being fed GM rapeseed. DNA was extracted from both earthworms and the rearing soil, and PCR was conducted to detect the *pat* gene. No amplification bands corresponding to the *pat* gene were observed in any replicate (Figure 7). These results suggest that horizontal gene transfer from GM rapeseed to earthworms or soil microorganisms is unlikely.

## Discussion

GM crops have been widely adopted in agriculture because of their agronomic advantages, including herbicide resistance, pest tolerance, and stress resilience.^14^ However, concerns remain regarding their potential environmental risks, particularly their impact on non-target organisms and soil ecosystems. Among these concerns, potential alterations of gut microbial communities and horizontal gene transfer from GM crops to soil organisms have attracted substantial attention.^15,16^

In this study, we assessed the potential impact of herbicide-resistant GM rapeseed expressing the *pat* gene on the gut bacterial diversity of *E. fetida*, a key soil invertebrate. Earthworms play crucial ecological roles in soil turnover and microbial regulation, making them ideal models for evaluating the biological effects of GM crop residues on the soil environment. To investigate the potential effects of GM rapeseed ingestion, we analyzed the gut microbiota composition of earthworms fed either GM or non-GM rapeseed and examined the presence of the inserted *pat* gene in both earthworm tissues and rearing soil.

Our results demonstrate that feeding GM rapeseed did not result in any significant alterations in the bacterial community structure or diversity of the gut microbiota of *E. fetida*. This is consistent with previous studies involving other GM crops, such as *Bt* maize or herbicide-resistant maize, which indicated negligible effects on rhizosphere bacterial communities under field or controlled conditions.^17–20^ Similarly, in mammalian models, including pigs and *Macaca fascicularis*, long-term feeding with GM crops showed no biologically significant changes in gut microbiota or associated health parameters.^7,21,22^

Furthermore, to assess the possibility of horizontal gene transfer from GM rapeseed to earthworms or associated soil microbes, we performed PCR targeting the *pat* gene. No amplification was observed in any of the three replicates from either earthworm body or rearing soil samples. These findings are consistent with previous reports that failed to detect gene transfer from GM plants to soil bacteria under natural or semi-natural conditions.^12,23^ Although horizontal gene transfer has been observed under highly artificial laboratory conditions, the frequency is extremely low and often not biologically relevant.^24^ Our results provide additional evidence that gene transfer from GM crops to soil invertebrates or microbial communities is unlikely under realistic exposure scenarios.

Notably, although some studies have reported statistical differences in physiological or hematological parameters following GM crop ingestion,^25,26^ these variations are often not dose-dependent or biologically significant. Similarly, in our study, the lack of changes in microbial diversity and the absence of detectable transgene presence in soil ecosystems suggest that GM rapeseed expressing *pat* does not pose a measurable risk to soil fauna or associated microbial communities.

Nevertheless, the interpretation of microbiome data can be influenced by methodological factors, such as DNA extraction efficiency, primer selection for 16S rRNA amplification, and bioinformatic processing.^27^ Although our study employed standard and validated protocols, these technical limitations should be considered in future studies.

## Conclusion

Our findings indicate that herbicide-resistant GM rapeseed does not significantly affect the gut bacterial diversity of *E. fetida* and does not result in detectable horizontal transfer of the *pat* gene to earthworms or the rearing soil microbiota. These findings support the environmental safety of GM rapeseed cultivation in terms of soil invertebrates and microbial integrity. However, long-term field studies across diverse soil types and climatic conditions are essential to further substantiate the ecological safety of GM crop cultivation.
